# Sutureless Aortic Valve Prosthesis in Redo Procedures: Single-Center Experience

**DOI:** 10.3390/medicina59061126

**Published:** 2023-06-11

**Authors:** Alina Zubarevich, Eleftherios T. Beltsios, Arian Arjomandi Rad, Lukman Amanov, Marcin Szczechowicz, Arjang Ruhparwar, Alexander Weymann

**Affiliations:** 1 Department of Thoracic and Cardiovascular Surgery, West German Heart and Vascular Center, University of Duisburg-Essen, Hufelandstraße 55, 45122 Essen, Germany; 2 Department of Cardiothoracic, Transplant and Vascular Surgery, Hannover Medical School, 30625 Hannover, Germany; 3 Medical Sciences Division, University of Oxford, Oxford OX3 9DU, UK

**Keywords:** sutureless aortic valve, Perceval, SU-AVR, Redo SU-AVR, reoperations, redo, reoperative aortic valve replacement

## Abstract

*Background and Objectives*: Sutureless aortic valve prostheses have presented favorable hemodynamic performance while facilitating minimally invasive access approaches. As the population ages, the number of patients at risk for aortic valve reoperation constantly increases. The aim of the present study is to present our single-center experience in sutureless aortic valve replacement (SU-AVR) in reoperations. *Materials and Methods*: The data of 18 consecutive patients who underwent SU-AVR in a reoperation between May 2020 and January 2023 were retrospectively analyzed. *Results*: The mean age of the patients was 67.9 ± 11.1 years; patients showed a moderate-risk profile with a median logistic EuroSCORE II of 7.8 (IQR of 3.8–32.0) %. The implantation of the Perceval S prosthesis was technically successful in all patients. The mean cardiopulmonary bypass time was 103.3 ± 50.0 min, and the cross-clamp time was 69.1 ± 38.8 min. No patients required a permanent pacemaker implantation. The postoperative gradient was 7.3 ± 2.4 mmHg, and no cases of paravalvular leakage were observed. There was one case of intraprocedural death, while the thirty-day mortality was 11%. *Conclusions*: Sutureless bioprosthetic valves tend to simplify the surgical procedure of a redo AVR. By maximizing the effective orifice area, sutureless valves may present an important advantage, being a safe and effective alternative not only to traditional surgical prostheses but also to transcatheter valve-in-valve approaches in select cases.

## 1. Introduction

For more than 50 years, surgical aortic valve replacement (SAVR) has been a gold-standard procedure for aortic valve disease, with complications and mortality continuously decreasing in recent years [1]. The growing population of elderly patients requiring an aortic valve replacement highlights the significance of implementing minimally invasive approaches [2,3]. It has been reported that more than half of the patients receiving a bioprosthesis need a reoperation in a time frame of 20 years after the primary operation [4]. As the population ages, the number of patients at risk for aortic valve reoperation (redo SAVR) is constantly increasing [4,5,6,7]. Unfavorable results after conventional SAVR with older generations of biological valve prostheses and the tendency towards the unitization of biological prostheses in younger patients with the idea of preforming a valve-in-valve transcatheter aortic valve replacement (ViV-TAVR) in the future have contributed to the higher rates of redo aortic valve procedures over the last decade [8]. Redo surgeries are commonly considered to be associated with a higher operative risk [6,9]. In fact, redo SAVR may be a challenge for the surgeon and is usually correlated with higher rates of mortality and complications [5,10].

Transcatheter aortic valve replacement (TAVR) is shown to have favorable survival results in high-risk patients with aortic valve disease compared to conventional SAVR and is reported to be at least as safe as SAVR in lower-risk patients [11,12,13]. Furthermore, TAVR offers the possibility for ViV-TAVR treatment in the cases of degenerated primarily surgically implemented bioprosthetic valves. Rising as a promising alternative to redo-SAVR, ViV-TAVR has been found to be comparably effective to SAVR while presenting a shorter hospital stay. Nevertheless, there is evidence that ViV-TAVR has higher rates of severe patient–prosthesis mismatch (PPM) and paravalvular leakage [14], a higher rate of readmission [15], and inferior postoperative gradients in comparison to redo-SAVR [14,16]. With TAVR becoming very popular in the latest years, surgical techniques are moving towards minimally invasive strategies.

Sutureless aortic valves have been found to decrease the overall operative time, cardiopulmonary bypass (CPB) time, and aortic cross-clamp (CC) time [17] while facilitating minimally invasive approaches such as non- or partial sternotomy minimally invasive AVR [18]. By eliminating the sewing ring at the valve’s base, the effective orifice area is maximized, resulting in a significant improvement in postoperative gradients [19,20]. Sutureless valves may present a considerable advantage over conventional redo-AVR due to their design properties and easy implementation. The most appropriate approach for patients with degenerated bioprostheses requiring redo-AVR remains a subject under investigation [9], as there is only limited evidence in the literature regarding the outcomes of SU-AVR in redo-AVR. The aim of the present study is to present our single-center experience in sutureless aortic valve replacement (SU-AVR) in reoperative procedures.

## 2. Materials and Methods

### 2.1. Study Design

The present study is a nonrandomized, retrospective, single-center study including 18 patients who, between May 2020 and January 2023, underwent redo SU-AVR at our institution. We analyzed postoperative outcomes and complications of patients undergoing redo SU-AVR in isolated and combined procedures using the Perceval S aortic valve prosthesis (Corcym, Saluggia, Italy).

### 2.2. Inclusion Criteria

Every patient who underwent redo SU-AVR using the Perceval S valve with or without concomitant procedures was eligible for this study. Patients undergoing redo procedures presenting with infective endocarditis were not seen as a contraindication for Perceval valve implantation. Patients presenting with bicuspid aortic valves or severely calcified ascending aorta were also excluded from the study. All cases were evaluated preoperatively by our institutional interdisciplinary Heart Team, consisting of a cardiac surgeon, cardiac anesthesiologist, and interventional cardiologist.

### 2.3. Operative Techniques

A median resternotomy was performed to access the chest. During the procedure, the heart was maintained in a normothermic state of cardiac arrest. The CPB was initiated with the direct cannulation of the ascending aorta and right atrium, with the exception of the cases where bicaval cannulation was required for tricuspid valve procedures. Custodiol HTK, a product manufactured by Köhler Chemie GmbH in Bensheim, Germany, was administered either through the aortic route or directly into the coronary ostia in cases of severe aortic regurgitation. The prosthetic or native aortic valve was then removed in its entirety through a high transverse aortotomy.

The Perceval S sutureless aortic valve was implanted following the positioning of three 4.0 prolene guiding sutures in the middle of each nadir. The implantation was carried out using the Snugger technique as previously described [21].

The aortotomy was closed using a 4.0 prolene pledgeted double-layered suture. After the performance of the prosthetic valves was evaluated and after deairing the heart, the CPB support was discontinued. Anticoagulation was completely reversed, and the chest was closed with steel wires in a conventional manner after ensuring that hemostasis was secured.

### 2.4. Concomitant Procedures

In patients undergoing concomitant coronary artery bypass surgery (CABG), the left internal thoracic artery was harvested using the pedicled technique. After carrying out distal coronary anastomoses in a standard manner, proximal coronary anastomoses, if necessary, were performed during cardiac arrest without additional clamping of the aorta to avoid damaging the sutureless prosthesis.

Tricuspid valve annuloplasty was performed using a Duran AnCore Annuloplasty Band (Medtronic, Dublin, Ireland) on the beating heart during total cardiopulmonary bypass prior to administering the cardioplegic solution.

For all mitral valve procedures, the mitral valve was accessed through left atriotomy via Waterston’s groove. For mitral valve repair, we used the Memo 4D Annuloplasty Ring (CORCYM, Saluggia, Italy), and, for mitral valve replacement, Medtronic Hancock II (Medtronic, Dublin, Ireland) bioprosthesis was used. In the case of mitral valve replacement, care was taken with regards to the exact positioning of the struts of the valve bioprosthesis to avoid the obstruction of the left ventricular outflow tract as reported in our previous research [3]. One of the struts was positioned halfway between the medial and lateral fibrous trigons of the mitral valve annulus away from the aortomitral continuity to avoid compromising the left ventricular outflow tract. The left atriotomy was closed with 4.0 prolene running suture prior to implantation of the Perceval prosthesis.

### 2.5. Data Collection

Prospective data were collected for our institutional database, including patient demographics, clinical characteristics, laboratory results, echocardiographic data, hemodynamic parameters, intraoperative variables, and postoperative outcomes. This study adhered to the 2013 revised Declaration of Helsinki and received approval from our institutional ethics board (approval #21-10350-BO), waiving individual patient consent. Patients provided signed informed consent for follow-up visits at the hospital. All methods were performed in accordance with the regulations and guidelines.

### 2.6. Outcomes and Definitions

The primary endpoints of this study were 30-day mortality and technical device success, which was evaluated through transthoracic echocardiography. The secondary endpoint was the development of any postoperative adverse events as defined by the Valve Academic Research Consortium (VARC-2).

Urgent procedures were defined as procedures which had to be performed in the same in-hospital stay. Emergent procedures were defined as procedures which had to be performed within the next 24 h.

### 2.7. Statistical Analysis

The obtained data were entered into a dedicated Microsoft Excel spreadsheet. Statistical analysis was performed using IBM SPSS version 27 (IBM Corp., Chicago, IL, USA). Data were tested for normality using the Shapiro–Wilk test. When data were not normally distributed, continuous variables were expressed as medians (interquartile range, IQR) or as means ± standard deviations. Categorical variables were expressed as frequencies and percentages. 

## 3. Results

### 3.1. Baseline Characteristics

A total of 18 consecutive patients with a history of cardiac surgery presenting with multiple comorbidities were enrolled in the study. The mean age was 67.9 ± 11.1 years. Their baseline characteristics and demographics are shown in Table 1. Overall, the patients showed a moderate-risk profile with a median logistic EuroSCORE II of 7.8 (IQR of 3.8–32.0) %. Sixteen patients (88.8%) had previously undergone an open cardiac surgery via median sternotomy, and two patients had been treated via left lateral thoracotomy. A total of fourteen patients (77.8%) had previously undergone a prosthetic aortic valve replacement. A total of ten patients (55.6%) showed a preoperative impaired renal function, and five patients (27.8%) presented with acute infective endocarditis.

### 3.2. Intraoperative Characteristics

An isolated redo SU-AVR was performed on 11 (61.1%) patients, concomitant CABG was performed on 1 (5.6%) patient, and 6 patients (33.3%) underwent a multivalve procedure involving SU-AVR. The implantation of the Perceval S prosthesis was technically successful in all the patients without any intraprocedural complications. No left ventricular outflow tract obstruction occurred; no second valve was required (Table 2). The overall procedural time (skin-to-skin) was relatively short, averaging at 158.7 ± 73.3 min; the mean CPB time was 103.3 ± 50.0 min; and the CC time for all the procedures was 69.1 ± 38.8 min.

### 3.3. Postoperative Outcomes and Survival

The median follow-up time for the whole cohort was 248.5 (IQR of 37.5–462.25) days. None of the patients required re-exploration for bleeding. No patients suffered postoperative AV-Block III°, requiring a permanent pacemaker implantation. We observed no cases of clinical- and/or imaging-confirmed stroke in our cohort. The postoperative aortic valve mean pressure gradient was 7.3 ± 2.4 mmHg. We observed no cases of paravalvular leakage. There was one case of intraprocedural death due to severe vasoplegia in a patient presenting with septic shock due to acute infective prosthesis endocarditis. The thirty-day mortality was 11%. There were no cases of early reoperations due to infective endocarditis at follow-up (Table 3).

Table 4 shows the Perceval sizes for each explanted sutured aortic valve prosthesis and their true inner diameter.

## 4. Discussion

The implementation of minimally invasive techniques and a reduction in ischemia–reperfusion injuries are goals that are of high importance for cardiovascular surgery in the era of transcatheter interventions. The aging population of patients presenting with multiple comorbidities highlights the importance of the minimization of surgical trauma and the operative time [2]. In this regard, both TAVR and SU-AVR are considered to be safe and effective less invasive approaches compared to conventional SAVR [3,22,23]. It is shown that sutureless valves achieve a significant reduction in CPB time, CC time, and overall operative time, while excellent hemodynamic results and a low rate of paravalvular leakage are reported [24]. This is highly significant, as it not only enables the possibility of a larger ViV-TAVR valve in the future but also significantly reduces gradients when replacing degenerated surgical valves [20]. Although still considered to be an off-label use, the utilization of sutureless aortic valve prostheses in reoperative aortic valve procedures has been described in multiple case reports and smaller single- and multicenter trials as a feasible and effective surgical method [10,20,25]. In this study, we presented our single-center experience in SU-AVR in redo procedures.

In the review article of Vendramin et al. [17], it is reported that, in groups of patients with failing stentless bioprostheses replaced by sutureless bioprostheses, the mean CPB time was 112 ± 50 min and that the mean aortic cross-clamp time was 59 ± 17 min. In our cohort, the mean overall procedural time (skin-to-skin) was 158.7 ± 73.3 min; the CPB time was lower, averaging at 103.3 ± 50.0 min; and the CC time for all the procedures was 69.1 ± 38.8 min.

In our study, none of the patients required re-exploration for bleeding. No patients developed AV-Block III° postoperatively, requiring a permanent pacemaker implantation. These results are encouraging when taking into consideration the previously reported rates of permanent pacemaker implantation following SU-AVR [26]. It is of importance to re-emphasize the significance of avoiding oversizing to reduce the mechanical stress on the atrioventricular node [18].

In our cohort, we reported a relatively high rate of new-onset dialysis (22.2%). However, over half of our patients (55.6%) preoperatively presented with chronic kidney function impairment, and, taking into consideration that 27.8% of the patients suffered from active infective endocarditis as well as considering that the median EuroSCORE II in our cohort was 7.8%, it is not surprising that the dialysis rate was high. In their meta-analysis, Nalluri et al. showed that the rates of postoperative kidney failure treated with dialysis were not significantly different between SAVR and valve-in-valve TAVR [27]. Interestingly, none of our patients with preoperatively normal kidney function suffered any relevant acute kidney injury postoperatively.

Neurological complications, such as stroke, are major concerns both in TAVR and in redo-SAVR. Stroke rates as high as approximately 9.7% for both redo-SAVR and ViV-TAVR were previously reported in the literature [20]. Dhanekula et al. reported concerning neurological outcomes in the early patients of their cohort, but improved outcomes were shown by acquiring further experience with these types of devices. This highlights that, although sutureless valves are designed to simplify the procedure, an important “learning curve” still exists with the use of these prostheses [20]. No cases of imaging-confirmed stroke were reported in our cohort. This could be attributed to the increasing learning curve of the surgeons performing the procedures, as our center has gained substantial experience with SU-AVR over the last few years. Additionally, all proximal CABG anastomoses were performed in the CC time, avoiding any additional manipulation of the aorta. We postoperatively observed no cases of myocardial infraction, and acute kidney injury requiring temporary dialysis occurred in four (22.2%) of the patients. Knowing that 55.6% of the cohort was initially suffering from a relevant renal function impairment, it may be considered to be an expected complication. Furthermore, both redo-SAVR and ViV-TAVI have been linked with high rates of postoperative AKI (15.2 ± 9.6% for redo SAVR) [28], with the cause being multifactorial.

The postoperative aortic valve mean pressure gradient was 7.3 ± 2.4 mmHg in our cohort, and we observed no cases of paravalvular leakage. Proper sizing and, particularly, avoiding oversizing are of high importance for the successful implementation of sutureless valves and for the elimination of postoperative complications. In fact, it is widely stated that correct sizing correlates with less paravalvular leakage, reduced peak and mean gradients, and lower permanent pacemaker implantation rates [29,30].

In our study, we reported no severe valve-related procedural complications, and the implantation of the Perceval S prosthesis was technically successful in all the patients. No left ventricular outflow tract obstruction occurred; no second valve was required. The thirty-day mortality was 11%, while there was one case of intraprocedural death due to severe vasoplegia in a patient presenting with acute septic shock due to acute infective prosthesis endocarditis and a EuroSCORE II rate of 35.2%. There were no cases of an early reoperation due to infective endocarditis at follow-up time, which was 248.5 (IQR of 37.5–462.25) days. The relatively high mortality is explainable by the fact that, in the cases which contributed to the high mortality, SU-AVR was used as a bail-out option, and, although we reported technically successful implantation, the patients still carried a high mortality risk due to their preoperative clinical state.

Reoperative cardiac surgery is commonly considered to be associated with an increased operative risk [6,9], while there is a substantial variability in reported surgical mortality rates with regards to redo-SAVR over the recent years, which might be attributed to differences in the risk profile of the patients and surgical experience [6,31]. In fact, this has further contributed to the recent popularization of TAVR in patients who were previously operated on and who present with a new onset of native aortic valve disease as well as of ViV-TAVR in the case of bioprosthetic degeneration [5,15,32,33]. Nevertheless, there is evidence that redo-SAVR in experienced centers presents overall satisfactory results and mortality rates, especially for patients with no endocarditic etiology and for patients without severely depressed left ventricles, or those not of preoperative NYHA functional class IV [5]. Interestingly, due to the limited durability of the first generations of bioprosthetic valves, there is substantial experience in many centers with regards to redo-SAVR in patients with degenerated bioprostheses [34,35]. Particularly, in elective cases, replacing a failing bioprosthesis is not considered to be extremely technically challenging amongst experienced surgeons [17]. Amongst the factors that contribute to the increased difficulty of redo-SAVR is the fact that the annulus of the aortic valve is often severely stiff, narrowed, and calcified. Under these conditions, the positioning of the anchoring stitches and the implementation of a stented prosthesis of an adequate size is challenging [17]. Due to their easy implementation and their design, which maximizes the effective orifice area, sutureless valves may have a significant advantage compared to conventional redo SAVR or ViV-TAVR [20]. Although the 30-day mortality rate is found to be lower in ViV-TAVR when compared to redo-SAVR, this advantage is considered to disappear between 30 days and 1 year; meanwhile, 1 year after the operation, it is reversed, favoring redo-SAVR [36].

Dhanekula et al. (16) investigated the relationship between the size of the explanted valve prostheses and the size of the implanted sutureless valve prostheses [20]. They found that the implanted prostheses were larger than the explanted ones, and this was also observed in our cohort, except for in one patient who had a previous full root replacement with a Biointegral prosthesis. This increase in valve size undoubtedly contributed to the favorable hemodynamic performance that was confirmed by the postoperative echocardiography. In their case report, Falcetta et al. [37] described the specifics of the utilization of a sutureless aortic valve prosthesis in patients who had previously undergone a full root replacement with a homograft. Similar to their report, Vendramin et al. [17] emphasized the advantages of sutureless prostheses in reoperative aortic root surgery in which the favorable larger inner diameter may contribute to the easier implantation into the previously implanted aortic valve without the reimplantation of the coronary buttons. In our cohort, we successfully performed a redo SU-AVR on a patient with a previously implanted Biointegral aortic root prosthesis the same way as described by Vendramin et al. [17].

Despite the fact that SU-AVR in patients with infective endocarditis is still considered to be an off-label use in Europe, five patients (27.8%) in our cohort presented with infective prosthesis endocarditis. In their studies, Sponga et al. and Lio et al. reported the successful implementation of SU-AVR in patients presenting with infective endocarditis [38,39], and our study group also previously published on this related topic [40].

### Study and Clinical Limitations

This study had some limitations, such as being retrospective and nonrandomized in design, coming from only one center, and having a limited number of patients. This may have influenced the results, reduced the study’s power, and increased the possibility of bias. Previous research on this topic has only been conducted on small mainly single-center groups, and further larger-scale prospective studies are necessary to validate the method’s safety and effectiveness. In the context of redo-AVR where a previous biological prosthesis was in place, the implementation of a Perceval sutureless valve may not be feasible in the cases of severe mitral valve disease, complex aortic root anatomy, a small aortic annulus, and the presence of bulky calcifications or fibrotic tissue around the previous prosthesis. These anatomical limitations may prevent the proper anchoring and sealing of the new prosthesis, increasing the risk of periprosthetic leakage, valve migration, or even structural valve failure. Therefore, a thorough preoperative evaluation and the careful selection of patients are essential to ensure the feasibility and safety of the procedure. In cases where the Perceval valve is not feasible, alternative surgical techniques, such as root replacement, stentless valve implantation, or valve-in-valve transcatheter aortic valve replacement, may be considered.

## 5. Conclusions

Sutureless aortic valves have gained considerable attention in recent years. Owing to its superior hemodynamic performance; decreased operative, CC, and CPB times; and their innovative design, SU-AVR has been established as a promising alternative for both traditional SAVR and TAVR procedures. SU-AVR has emerged as a tailored surgical concept for patients who are ineligible for ViV-TAVR as well as a valuable tool for implementing bailout strategies in high-risk patients.

In this single-center study, we reported from a unique perspective on sutureless prosthesis application in redo procedures that was guided by the seasoned proficiency of our surgical team. Although there are few other studies on the implementation of sutureless aortic valve prostheses in redo procedures, the data on this matter still appear scarce, and this study not only bolsters the existing knowledge but also introduces fresh insights by showcasing consistent techniques, revealing potential off-label uses, and sharing the insights of an experienced surgical team.

Although redo SU-AVR is proven to be a safe and feasible approach, larger trials should follow to further investigate the long-term safety and efficacy of these devices in reoperative cases.

## Figures and Tables

**Table 1:** Baseline characteristics.

Age	67.9 ± 11.1
BMI, kg/m^2^	27.5 ± 3.4
Atrial fibrillation	7 (38.9%)
Arterial hypertension	16 (88.9%)
Pulmonary hypertension	3 (16.7%)
Diabetes mellitus type 2	4 (22.2%)
Chronic obstructive pulmonary disease	3 (16.7%)
Coronary artery disease	12 (66.7%)
Chronic kidney injury	10 (55.6%)
Creatinine, g/dL	1.7 ± 1.5
Infective endocarditis	5 (27.8%)
Previous operation:	
SAVR	12 (66.7%)
Bentall	1 (5.6%)
CABG	5 (27.8%)
David	1 (5.6%)
SMVR	2 (11.1%)
STVRp	1 (5.6%)
PDA closure	1 (5.6%)
TA-TAVR	1 (5.6%)
EuroSCORE II, %	7.8 (IQR of 3.8–32.0)
Time since the first operation, days	1561.5 (IQR of 1232.7–3504.0)
Time since the first operation, years	4.28 (IQR of 3.8–9.6)
AV-MPG, mmHg	35.6 ± 18.2
AR moderate or greater	7 (38.9%)
AS moderate or greater	9 (50%)
MS moderate or greater	1 (5.6%)
MR moderate or greater	5 (27.8%)
TAPSE, mm	20.8 ± 3.0
EF, %	47.9 ± 12.7

AR—aortic regurgitation, AS—aortic stenosis, AV—aortic valve, BMI—body mass index, CABG—coronary arterial bypass grafting, EF—ejection fraction, PDA—patent ductus arteriosus, SAVR—surgical aortic valve replacement, SMVR—surgical mitral valve replacement, STVRp—surgical tricuspid valve repair, TA-TAVR—transapical transcatheter aortic valve replacement, and TAPSE—tricuspid annular plane systolic excursion.

**Table 2:** Intraoperative characteristics.

Procedural Time, min	158.7 ± 73.3
CPB time, min	103.3 ± 50.0
CC time, min	69.1 ± 38.8
Urgency of the procedure	
elective	7 (38.9%)
urgent	8 (44.4%)
emergent	3 (16.7%)
Concomitant procedure	
CABG	1 (5.6%)
SMVR	4 (22.2%)
SMVRp	2 (11.1%)
TMVRp	2 (11.1%)
Myectomy	1 (5.6%)
Perceval size	
S (21 mm)	2 (11.1%)
M (23 mm)	7 (38.9%)
L (25 mm)	3 (16.7%)
XL (27 mm)	6 (33.3%)

CABG—coronary arterial bypass grafting, CC—cross-clamp, CPB—cardiopulmonary bypass, SMVR—surgical mitral valve replacement, SMVRp—surgical mitral valve repair, and TMVRp—surgical tricuspid valve repair.

**Table 3:** Perioperative outcomes.

Postoperative MPG, mmHg	7.3 ± 2.4
Paravalvular leakage	0
30-day mortality	2 (11.1%)
In-hospital length of stay	11.4 ± 6.2
ICU length of stay	4.5 (IQR of 2.0–7.25)
New onset dialysis	4 (22.2%)
Stroke	0
New pacemaker implantation	0
Re-endocarditis at follow-up	0
Follow-up time, days	248.5 (IQR of 37.5–462.25)

ICU—intensive care unit; MPG—mean pressure gradient.

**Table 4:** Prosthesis sizes prior to and after redo procedure.

Valve Type	Size, mm	Perceval Size	ID of Explanted Valve, mm	ID of Implanted Valve, mm
Perimount	23	M	22	23
Biointegral	29	M	29	23
Native AV		S		21
Perimount	23	XL	22	27
SJM Biocor	21	S	19	21
Native AV		XL		27
Perimount	21	M	20	23
Perimount	23	L	22	25
Trifecta GT	25	XL	22	27
Perimount	23	XL	22	27
Perimount	21	M	20	23
Perimount	23	L	22	25
Sapien	26	L	23	25
Trifecta	23	M	21	23
Native AV		M		23
Perimount	23	XL	22	27
Native AV		XL		27
Perimount magna ease	19	M	18	25

AV—aortic valve; ID—inner diameter.

## Data Availability

Data are available on request from the authors. The data that support the findings of this study are available from the corresponding author upon reasonable request.
